# Metals in Alzheimer’s Disease

**DOI:** 10.3390/biomedicines11041161

**Published:** 2023-04-12

**Authors:** Mirjana Babić Leko, Lea Langer Horvat, Ena Španić Popovački, Klara Zubčić, Patrick R. Hof, Goran Šimić

**Affiliations:** 1 Department of Neuroscience, Croatian Institute for Brain Research, University of Zagreb School of Medicine, 10000 Zagreb, Croatia; 2 Nash Family Department of Neuroscience, Friedman Brain Institute and Ronald M. Loeb Center for Alzheimer’s Disease, Icahn School of Medicine at Mount Sinai, New York, NY 10029, USA

**Keywords:** Alzheimer’s disease, essential metals, heavy metals, biomarker, Mendelian randomization

## Abstract

The role of metals in the pathogenesis of Alzheimer’s disease (AD) is still debated. Although previous research has linked changes in essential metal homeostasis and exposure to environmental heavy metals to the pathogenesis of AD, more research is needed to determine the relationship between metals and AD. In this review, we included human studies that (1) compared the metal concentrations between AD patients and healthy controls, (2) correlated concentrations of AD cerebrospinal fluid (CSF) biomarkers with metal concentrations, and (3) used Mendelian randomization (MR) to assess the potential metal contributions to AD risk. Although many studies have examined various metals in dementia patients, understanding the dynamics of metals in these patients remains difficult due to considerable inconsistencies among the results of individual studies. The most consistent findings were for Zn and Cu, with most studies observing a decrease in Zn levels and an increase in Cu levels in AD patients. However, several studies found no such relation. Because few studies have compared metal levels with biomarker levels in the CSF of AD patients, more research of this type is required. Given that MR is revolutionizing epidemiologic research, additional MR studies that include participants from diverse ethnic backgrounds to assess the causal relationship between metals and AD risk are critical.

## 1. Alzheimer’s Disease

Alzheimer’s disease (AD) is the most common cause of dementia worldwide (60–70% of cases), affecting over 55 million people. It is predicted that 74.7 million people will have dementia by 2030 and approximately 131.5 million by 2050. By 2030, the cost of treating and caring for dementia patients will rise to USD 2 trillion (http://www.worldalzreport2015.org/ accessed on 22 February 2023).

The accumulation of two proteins, amyloid beta (Aβ) and tau, in amyloid plaques and neurofibrillary tangles (NFTs), respectively, is thought to contribute to the development and progression of AD. Although the precise relationship between these two pathologies is unknown, there is evidence that they may interact in a bidirectional manner to promote each other’s aggregation and toxicity [1]. Overall, the relationship between Aβ and tau in AD is complex, with multiple feedback loops and interactions with other pathological processes [2].

Aβ is a small peptide derived from the amyloid precursor protein (APP). APP is a transmembrane protein with an unknown function in the brain, but it is thought to be involved in cell adhesion, signaling, and synapse formation [3]. Different enzymes can cleave APP, resulting in the production of a number of peptides of variable lengths. β-Secretase cleaves APP at the N-terminus of the Aβ domain in the amyloidogenic pathway, followed by γ-secretase cleavage within the transmembrane domain, resulting in the production of Aβ peptides with varying lengths [4]. Excessive Aβ formation, aggregation, and deposition in the brain are thought to result in the formation of amyloid plaques [5]. Aβ forms insoluble oligomers and even larger insoluble fibers when it aggregates. The longer form of the peptide (Aβ_1–42_) promotes abnormal Aβ peptide aggregation more strongly than the shorter form (Aβ_1–40_) [6]. The amyloid build-up is thought to activate microglia and cause an inflammatory response [7]. Aβ can also accumulate in the walls of meningeal and cerebral arteries, arterioles, capillaries, and veins, causing a condition known as cerebral amyloid angiopathy (CAA) [8,9,10,11].

Tau, on the other hand, is a microtubule-associated protein found primarily in the axons of healthy neurons. In this context, tau is a critical regulator of microtubule dynamics, modulating their assembly, elongation, and maturation. Tau helps stabilize microtubules and keep them aligned, which is necessary for neuronal function and transport of essential molecules and organelles [12]. Tau regulates the length, stability, and thickness of axonal microtubules by cross-linking of α and β tubulin monomers [13].

The underlying mechanisms that precede the formation of amyloid plaques and NFTs in the brains of AD patients as well as the relationships between these pathological lesions are not well understood [14,15]. The toxicity of Aβ deposits and mechanical damage to axons that impair axoplasmic transport, resulting in axonal sprouting to bridge the damaged portion of the axon, are two of the proposed mechanisms [16,17]. Axon sprouts and their microtubules should become less stable for the sprouts to form synapses, which becomes possible once tau proteins detach from the microtubules. Tau undergoes numerous post-translational modifications in AD including phosphorylation, acetylation, and O-glycosylation [18]. The phosphorylation of tau causes it to undergo a change in conformation and separate from microtubules [19]. When hyperphosphorylated, tau detaches from microtubules, the axons also disintegrate, resulting in neuronal death [20]. It is possible, however, that tau cleavage or folding occurs first, followed by phosphorylation and detachment from the microtubules [21]. In addition to amyloid and tau-related mechanisms, oxidative stress, neuroinflammation, mitochondria, lysosomes, neurovascular, and cell cycle dysfunction all play important roles in the pathological process of AD [22,23,24,25].

Previous studies have revealed that the homeostasis of essential metals is altered in AD [26,27,28], where iron (Fe), zinc (Zn), and copper (Cu) are the essential metals most commonly associated with AD pathological changes. Heavy metal concentrations have also been found to rise in AD brains [29].

This review summarizes research on the role of essential and heavy metals in AD. We included human studies that (1) compared the metal concentrations in AD patients and healthy controls, (2) compared the metal concentrations with concentrations of cerebrospinal fluid (CSF) biomarkers in AD subjects, and (3) used a Mendelian randomization methodology (MR) to assess the involvement of essential metals in AD. Two independent researchers searched Medline using the following keywords: “Alzheimer’s disease”, “aluminum”, “arsenic”, “barium”, “cobalt”, “copper”, “cadmium”, “calcium”, “iron”, “lithium”, “lead”, “mercury“, “magnesium”, “molybdenum”, “manganese”, “nickel”, “potassium”, “selenium”, “sodium”, “strontium”, “thallium”, and “zinc”. The literature search was completed on 23 February 2023.

## 2. Molecular Mechanisms through Which Metals Contribute to Alzheimer’s Disease Pathology

**Figure 1:**
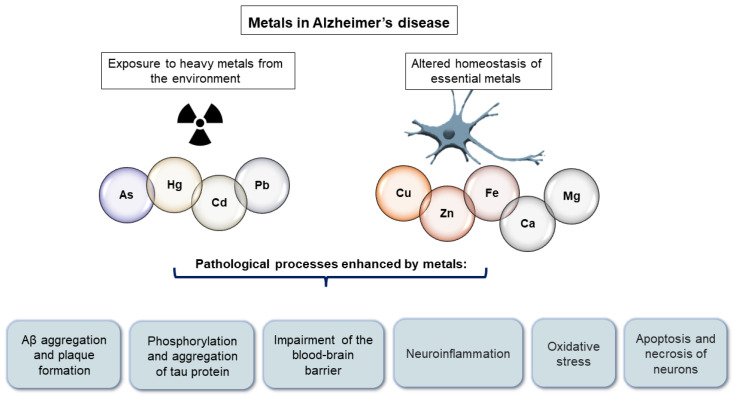
Pathological processes enhanced by metals in Alzheimer’s disease.

Increased metal concentration in the brain may contribute to various AD-associated pathological processes including Aβ-aggregation [30,31], hyperphosphorylation of tau protein [32,33], neuroinflammation [34], oxidative stress [35], blood–brain barrier (BBB) impairment [36], apoptosis and necrosis of neurons [37,38], and autophagy [39] (Figure 1). Experimental evidence indicates that both essential metals and heavy metals increase the aggregation of Aβ [30,40,41] and the hyperphosphorylation and aggregation of tau protein [33,42,43,44]. Furthermore, the exposure of young rats to a mixture of heavy metals induced neuroinflammation dependent on oxidative stress [45]. In addition, some essential metals such as Fe [46], Cu [47], Zn, and calcium (Ca) [39] can induce oxidative stress. Fe participates in Fenton reactions and can therefore contribute to the formation of reactive oxygen species [46]. Both the observed disruption of the BBB [48,49] and the apoptosis and necrosis of neurons [37,38] upon exposure to heavy metals may be preceded by oxidative stress, according to experimental evidence. Neurons are extremely sensitive to oxidative stress. Wang et al. [39] proposed that metal ion imbalance could induce oxidative stress, with the following downstream effects: (1) imbalance of protein kinases and phosphatases, increasing tau protein phosphorylation, and (2) imbalance of secretases, resulting in an increase in Aβ production (reviewed in [39]). On the other hand, essential metals also serve as cofactors in enzymes that combat oxidative stress. Cu, Zn, and manganese (Mn) are enzyme components of superoxide dismutase enzymes, while selenium is an enzyme component of glutathione peroxidase [50].

Although there is a substantial body of evidence linking metals to AD-related pathological processes, it is unclear whether disrupted metal homeostasis is involved in the pathogenesis of AD, results from AD pathological processes, or both. Given that AD is a complex disease driven by both genetic and environmental factors, it is unlikely that AD pathogenesis will be explained by a single factor, but rather by the interaction of many.

## 3. Heavy Metals in Alzheimer’s Disease

Heavy metals including arsenic (As) [51], cadmium (Cd) [49], lead (Pb) [52], and mercury (Hg) [52] can cross the BBB and accumulate in the brain, or they can bypass the BBB and enter the brain directly through the olfactory pathway [53]. Some researchers have hypothesized that early exposure to heavy metals is associated with the later development of AD. Based on their observations of experimental animals, they concluded that early-life exposure to As [54], Pb [55], and Cd [56] may contribute to the development of neurodegeneration later in life, which is consistent with the developmental hypothesis of AD [57,58,59].

### 3.1. Arsenic

As is a metalloid that can be ingested through contaminated water, soil, and air, but primarily through drinking contaminated water. More than 220 million people are estimated to consume water that exceeds the permissible level of 10 µg/L [60]. Epidemiological studies suggest that As contributes to cognitive impairment [61] and an increased risk of AD [62], and that elevated As levels in soil are associated with an increase in AD-related mortality [63]. As exposure has also been associated with memory impairments in animal studies [64,65,66]. As exposure also increases Aβ levels [67], promotes tau hyperphosphorylation [32,68,69], tau aggregation [32], oxidative stress caused mainly by mitochondrial dysfunction [70], vascular damage [71], neuroinflammation [34], and apoptosis and the necrosis of neurons [37,38] (Figure 1). In the majority of human studies, there were no significant differences in As levels between AD patients and the controls, although some studies observed a significant increase in As levels in AD patients [72,73] and a positive association with CSF AD biomarkers [74].

### 3.2. Cadmium

Humans are exposed to Cd through food, air, and water [75]. Smokers have Cd levels that are two to four times higher than nonsmokers [76]. Cd may also play a role in the development of AD pathological changes. Cd has been linked in human studies to increased mortality due to AD [77,78] and cognitive decline [79,80,81]. Ruczaj and Brzoska proposed that Cd primarily exerts its effects by inducing oxidative stress [82]. Nevertheless, it also interacts with Aβ [83] and increases Aβ aggregation [30,40], promotes tau hyperphosphorylation [33] and aggregation [42], impairs the BBB [48,49], impairs cholinergic transmission and causes the death of cholinergic neurons in the basal forebrain [84], and disrupts intracellular cation homeostasis by being an anti-metabolite of Zn and replacing it in Zn enzymes [85] (Figure 1). In human studies, there is either an increase [86] or no difference [87] in Cd levels between AD patients and healthy controls (Table 1).

### 3.3. Mercury

Exposure to Hg occurs through food, air, and water, with seafood consumption being the primary source of mercury poisoning [152]. Three- to 5-fold increases in Hg levels in the air and water have been documented as a result of industrialization [153]. A systematic review [154] and meta-analysis [155] demonstrated an association between Hg exposure and cognitive decline and progression of AD, but a subsequent report [156] did not confirm these findings. In addition, a neuropathological study of 286 brains by Morris et al. revealed no correlation between higher brain Hg levels and neuropathological alterations [152]. However, there are multiple molecular mechanisms through which Hg may contribute to the pathogenesis of AD. It promotes Aβ production [157] and aggregation [30], tau hyperphosphorylation [158,159] and aggregation [160], induces oxidative stress [35], and alters calcium homeostasis [161] (Figure 1). Human body fluid Hg measurements yielded contradictory results. Both an increase and a decrease were observed in Hg levels between the AD and control subjects, or there was no change (Table 1). In addition, the CSF Hg level was positively correlated with several CSF AD biomarkers [74], whereas the blood Hg level was positively correlated with the CSF Aβ_1–42_ level [145].

### 3.4. Lead

In addition to food, air, and water, humans are also exposed to lead [29] through ingestion. Epidemiological studies have demonstrated that lead exposure contributes to cognitive impairment [162,163]. Moreover, experimental studies have reported an association between Pb and AD pathological changes. Pb interacts with Aβ [31] and increases Aβ production [45,164] and aggregation [31], increases tau hyperphosphorylation [165], compromises the BBB [36], induces epigenetic modifications by altering the expression of AD-related genes [166,167], disrupts intracellular cation homeostasis by interfering with Ca homeostasis and replacing Zn ions in Zn enzymes [168], and induces oxidative stress [169]. In human studies, there was a decrease or no difference in the Pb levels between the AD patients and control subjects (Table 1), whereas a recent MR study found that higher blood Pb levels were a risk factor for AD [170].

### 3.5. Aluminum

Aluminum (Al), the most abundant metal in the Earth’s crust [171], is not an essential element for life; however, in its free, solvated, and trivalent forms, Al^3+^ is biologically reactive [172], accumulating in the central nervous system [173,174]. In AD-affected brain regions including the entorhinal cortex, hippocampal region, and amygdala, the concentration of Al is higher [175,176]. Al was co-deposited with fibrillar Aβ in amyloid plaques in a study of brain tissue samples from donors with familial AD (fAD) and the PSEN1-E280A (Glu280Ala) mutation [172,177]. Cortical Aβ levels are elevated in donors with this mutation, and this mutation is associated with an aggressive etiology of AD [178]. Aluminum’s unique association with Aβ and the high levels of Al found in these brain tissues suggest that Al plays a role in the neuropathology of fAD [177].

When Al binds to various proteins, oligomerization can occur, resulting in conformational changes that prevent proteases from degrading the proteins. In addition, Al^3+^ binds strongly to phosphorylated amino acids, causing highly phosphorylated cytoskeleton proteins to aggregate and accumulate [179]. As a result, Al induces the apoptotic death of neurons and glial cells. Al-Aβ co-deposition in fAD has been hypothesized, but its association with intraneuronal NFTs has not been confirmed [177,180], as demonstrated by Mold et al. [181]. While Al binding to Aβ in amyloid plaques is anticipated in the early stages of disease progression [177,178,182], an association with tau may occur in later disease stages [177,178,182]. Numerous studies have investigated the association between oral exposure to Al in drinking water and AD [183]. According to Martyn et al. [184], AD is more prevalent in regions with high levels of Al in their drinking water. In conclusion, even though Al has been proposed as a potential risk factor for AD, there is insufficient evidence to support a causal relationship (Table 2). Many studies have investigated the association between oral exposure to Al in drinking water and AD; however, more research is required to better understand how genetic, environmental, and lifestyle factors influence the onset and progression of AD.

## 4. Essential Metals in Alzheimer’s Disease

The homeostasis of essential metals is altered in AD patients [26,27,28]. This term refers to metals that are naturally present in the body and play a role in the function of numerous proteins and enzymes or act as second messengers. Sodium (Na), Ca, and magnesium (Mg) are the most abundant essential metals in the human body, while Fe, Cu, Zn, molybdenum (Mo), cobalt (Co), Mn, and chromium (Cr) are present in trace amounts. Many previous studies have also demonstrated the association between essential metals (primarily Fe, Cu, and Zn) and AD pathological changes.

### 4.1. Iron

Many biological processes in the body including the brain are regulated by Fe ions. Fe is essential for protein synthesis [197], cell growth and differentiation [198,199], the regulation of Fe-dependent enzymes [200], oxygen transport [201], and the electron transfer chain in oxidation–reduction reactions [201]. Fe is also crucial for the processes of myelination [202], development [203], and the function of numerous neurotransmitter systems [204]. Both amyloid plaques and NFTs have been found to have elevated Fe concentrations [205]. Fe is also involved in oxidative stress and the formation of reactive oxygen species in the brains of AD patients via the Fenton reaction [46]. Fe also promotes in vitro Aβ aggregation [206], tau protein phosphorylation [207,208,209], and tau aggregation [210] (Figure 1). It is interesting to note that APP is necessary for the persistence of ferroprotein (iron exporter) on the cell surface, and thus promotes Fe release [211].

In meta-analyses, a significant decrease in Fe levels was observed in the plasma [87] and serum [129] of AD patients, but no significant change was observed in the CSF [129] (Table 1). In contrast, a number of studies observed a correlation between the Fe levels in CSF and various CSF AD biomarkers [74,188,193] (Table 2). Nonetheless, in many observational studies, there was no difference in the Fe levels between the AD patients and controls (Table 1)..

### 4.2. Zinc

The brain has a higher Zn concentration than other organs [212]. Zn is essential for neurotransmission because, as an antagonist of glutamate NMDA (*N*-methyl-D-aspartate) receptors, it protects neurons from glutamate-induced excitotoxic damage [213]. Zn accumulates in amyloid plaques [214], binds to Aβ, and promotes Aβ aggregation and plaque formation [214]. Zn also promotes tau protein aggregation [215], phosphorylation [216,217], and translation [217] (Figure 1). In meta-analyses, however, a significant decrease in Zn levels was observed in the serum and plasma [123] as well as in the hair of AD patients [87], whereas there was no significant change in the CSF [123] and brain [115] levels (Table 1). To date, MR studies have not identified Zn as a risk factor for AD [218,219,220]. An in vivo study demonstrated positive effects of Zn supplementation in mouse models of AD [221], and a small double-blind clinical trial observed the stabilization of cognitive abilities in AD patients after six months [222]. Thus, adding Zn to the diet has been suggested to improve the cognitive abilities of AD patients [223], whereas Loef et al. found no significant benefit of Zn supplementation in AD [224]. In addition, in vivo studies have shown that Zn supplementation promotes the formation of NFTs [225] and Aβ deposition [226].

### 4.3. Copper

Normal brain function requires optimal Cu levels, as indicated by the disruption of its metabolism. Patients with Menkes syndrome, for example, suffer from intellectual deficits and neurodegeneration. This disorder is caused by a sex-linked mutation of the *ATP7A* gene on the X chromosome (which encodes a protein involved in the transmembrane transfer of Cu ions) and is characterized by the decreased absorption of Cu in the intestine, and consequently, a decreased concentration of Cu in the cytosol of all body cells except in the intestines and kidneys [227]. In Wilson’s disease, excessive Cu accumulation in the body is associated with psychosis, parkinsonism, and dementia [228,229]. Cu homeostasis is also impaired in AD [28]. Cu promotes the formation and accumulation of Aβ-oligomers by binding to Aβ [41]. Cu chelation can prevent the cytotoxic effect of the Cu-Aβ complex [230]. Cu accumulates in plaques [231,232], and the interaction between Cu and APP has been demonstrated [232]. Cu can induce both the phosphorylation and aggregation of tau [42,43] (Figure 1) and its interaction with apolipoprotein E (ApoE) contributes to the pathogenesis of AD. ApoE2 has the highest binding affinity for divalent Cu, Zn, and Fe ions, while ApoE4 has the lowest [233,234]. In meta-analyses, Cu levels in the serum of AD patients increased significantly [87,92,122,133], whereas Cu levels in the brains of AD patients decreased [92] (Table 1). Recent MR studies [218,220] have surprisingly found that higher Cu levels are protective against AD risk.

### 4.4. Calcium

Ca is an indispensable second messenger that regulates hundreds of signaling pathways crucial for the normal functioning of memory and cognition-related cells and networks [235]. Many neurodegenerative diseases including AD [236] are characterized by a disruption of cellular Ca signaling. The excessive entry of Ca ions through ionotropic glutamate receptors is a known mechanism of excitotoxic neuronal death [237,238]. Ca homeostasis disruption promotes Aβ and tau pathology [239]. However, human studies have produced contradictory results, with both decreased [240,241] and increased Ca [186,242] being risk factors. In recent MR studies, higher Ca levels were shown to reduce the risk of AD [241,243], or no association between Ca levels and AD risk has been observed [218,220] (Table 3).

### 4.5. Manganese

Mn is a crucial element for protein synthesis, lipid and glucose metabolism, and oxidative stress protection [244]. However, Mn is also an environmental toxin, and elevated Mn levels have been linked to diminished cognitive performance [187,245,246]. A rise in Mn levels has also been observed in patients with AD [109]. Nonetheless, a meta-analysis by Du et al. [89] revealed a significant decrease in Mn levels between AD and the controls.

### 4.6. Magnesium

Human studies have demonstrated that Mg deficiency impairs memory [247] and that Mg supplementation can improve memory in dementia patients [248,249,250]. In addition, a decrease in Mg concentration has been observed in the tissues of AD patients [251,252]. However, no change in Mg concentration was observed in the brains of AD patients in some studies (reviewed in [253]). Mg influences the processing and transport of APP, with low Mg levels favoring the β-secretase pathway and high Mg levels favoring the α-secretase pathway [254], whereas the treatment of experimental animals with Mg sulfate reduces tau phosphorylation and influences the maintenance of cognitive functions and synaptic plasticity [255]. According to the meta-analysis by Du et al. [89], the serum and plasma Mg concentrations were lower in the AD patients than in the controls, whereas the CSF Mg concentrations did not differ between groups. Thomassen et al. [95] did not find an association between the plasma Mg levels and the risk of AD in a study involving more than 100,000 participants. Kieboom et al. demonstrated that both low and high Mg concentrations were associated with an increased risk of dementia. They concluded that the relationship between Mg and the risk of dementia was U-shaped rather than linear [108].

### 4.7. Other Essential Metals

AD also perturbs the homeostasis of Na, K, and Co. Previous studies have associated elevated Na levels with AD [27,96,256,257]. Both increased [102] and decreased [195] K levels have been associated with AD, whereas in some studies, no change in the K levels was observed in AD. Co is an essential component of vitamin B_12_ and is an environmental toxin. Zheng et al. showed that mice exposed to Co develop age-related neurodegeneration [258].

## 5. Treatment of Alzheimer’s Disease Based on the Metal Hypothesis

So far, therapeutic interventions based on the metal hypothesis of AD have progressed in two directions. Taking metal supplements is one approach. As previously stated, there have been several clinical studies on the effect of Zn and Cu supplementation on cognitive performance [259]. Although it was previously thought that metal supplementation could delay the onset of dementia, the majority of studies have found no significant effect on cognitive function improvement [259]. Cu supplementation had no positive effects in a pilot phase 2 clinical trial in AD patients [260]. In contrast to this viewpoint, there is a theory about the therapeutic effect of chelating excess Zn, Cu, or Fe metals based on their ability to stimulate Aβ aggregation [261]. Chelators are substances that bind metals so that they cannot interact with the Aβ further. Based on promising results from in vivo studies on animal models [262], one of the compounds with chelating activity, clioquinol (CQ), has entered the second phase of clinical trials [263]. CQ can effectively bind Zn^2+^ and Cu^2+^ ions, and in vitro studies have shown that its effect can be achieved by stimulating neuroprotective signaling pathways by increasing the cellular uptake of Zn and Cu ions [264]. Furthermore, CQ can reduce the number of Aβ aggregates within the cells [264]. A clinical study found that the CQ had a promising effect on cognitive improvement, but only in patients with the worst starting point [263]. PBT-2, another similar substance, had a similar effect on AD patients. In a 2008 clinical trial, PBT-2 demonstrated an impact on Aβ metabolism by lowering the Aβ CSF concentrations, and a group of patients who received a higher dose of PBT-2 had better results in two of the executive functions tests, while other cognitive tests revealed no significant differences [265]. A recent study investigated the different capacities for chelating Cu from Cu(II)Aβ(1–42) complexes, and the results showed that CQ and B2Q were more efficient than PBT-2 [266], but it must be noted that when using such substances, they must be not too effective as chelators to disrupt the normal metal functions in the brain. Another recent in vivo study examining the effect of Cu ions on tau protein pathological changes found that lowering the Cu concentrations in the brain could help alleviate spatial memory deficits, but, neither lowering nor increasing the amounts of Cu affected the tau protein pathology [267]. A recent review study called the chelating theory into question on multiple levels [268]. As a result, there is no single metal-target approach for treating AD, and more research is needed to better understand this complex aspect of the disease.

## 6. Conclusions

In this review, we discussed the studies examining the role of essential metals and heavy metals in AD. Relevant studies involving human subjects were included. It is still challenging to paint a complete picture of how metals interact in AD pathogenesis because of the significant degree of variability in the results between studies. Cu and Zn showed the most consistent results, with most studies revealing that the AD patients’ Cu levels rose while their Zn levels fell (Table 1). However, several studies also failed to find such a link (Table 1). Comparing the metal levels with biomarkers from the AD subjects’ CSF has rarely been undertaken, and the results were sometimes contradictory (Table 2). The use of various methodologies to determine the metal levels and examine various body fluids may be the cause of the studies’ contradictory findings. Atomic absorption spectrophotometry (AAS) was the second most often employed method, while inductively coupled plasma mass spectrometry (ICP-MS) was the method of choice, being the method utilized in the majority of investigations into the metal measurements. Several MR studies have investigated the relationship between metals and the risk of AD (Table 3). Since MR is revolutionizing epidemiologic research [269], and given the importance of elucidating the role of metals in AD pathogenesis, additional MR studies examining the causal association between metals and AD risk and including people from various ethnic backgrounds are crucial.

## Figures and Tables

**Table 1:** Comparison of metal levels between dementia patients and healthy controls.

Reference	Analyzed Bodily Fluid	Method Used	Measured Metals	Classification of Participants (Number of Patients)	Number of Participants	Metals in AD Patients versus HC
[74]	CSF and plasma	ICP-MS	In CSF and plasma: As, B, Ca, Cd, Co, Cu, Fe, Hg, Li, Mg, Mn, Mo, Na, Ni, P, Pb, S, Se, Sr, Tl, Zn In CSF: Al, Ba, K	CSF: AD (124), MCI (50), HC (19) Plasma: AD (93), MCI (35), HC (15)	CSF: 193 Plasma: 143	In CSF: Zn↑ (*p* = 0.024), Al↓ (*p* = 0.003), P↑ (*p* = 0.029) In plasma: Na↑ (*p* = 0.004)
[87]	Bodily fluids	Meta-analysis	Cu, Fe, Zn, Se, Mn, Pb, Al, Cd, Cr, As, Hg, Co			In serum: Cu↑ (SMD [95% CI]); 0.37 (0.1, 0.65) In plasma: Fe↓ −0.68 (−1.34, −0.02), Se↓ −0.61 (−0.97, −0.25) In hair: Zn↓ −0.35 (−0.62, −0.08)
[88]	Serum		Al, Co, Cd, Cr, Cu, Fe, Mg, Mn, Se, Zn	Elderly with and without cognitive dysfunction	191	Cu↑ in elderly with cognitive dysfunction
[73]	Urine and blood	ICP-MS	In urine: As In blood: Cr and Se	AD (53), HC (217)	270	As↑ (*p* = 0.023), Cr↑ (*p* = 0.005), Se↓ (*p* = 0.001)
[89]	Serum, CSF	Meta-analysis	Mg	Serum and plasma: AD (1112), HC (1001) CSF: AD (284), HC (117)		In serum and plasma: Mg↓ (SMD [95% CI]); −0.89 (−1.36, −0.43)
[90]	CSF	ICP-MS	Fe, Ni, Cr, Zn, Mn, Co, Cu	AD (20), CAA (10), HC (10)	40	No difference
[27]	CSF and plasma	ICP-MS	Cu, Zn, Fe, Na, Mg, Ca, Co, Mo, Mn, B	CSF: AD (126), MCI (52), HC (19) Plasma: AD (93), MCI (37), HC (14)	CSF: 197 Plasma: 144	In CSF: Zn↑ (*p* = 0.027) In plasma: Na↑ (*p* = 0.004)
[72]	Hair and nail samples	ICP-MS	As, Se	AD (40), HC (40)	80	In hair and nail samples: As↑ (*p* < 0.001), Se↑ (*p* < 0.001)
[86]	Blood and serum	ICP-OES	In blood: Cd, Hg, Al, Pb, As In serum: Zn, Cu, Fe	AD (50), HC (50)	100	Cd↑ (*p* < 0.001), Hg↑ (*p* < 0.001), Al↑ (*p* = 0.009), Cu↑ (*p* = 0.025), Fe↓ (*p* = 0.030), Zn↓ (*p* < 0.001)
[91]	Serum	AAS	Cu, Zn, Se	AD (110), HC (60)	170	Se↓ (*p* < 0.05), Zn↓ (*p* < 0.001), Cu/Se↑ (*p* < 0.001)
[92]	Serum, plasma, and brain	Meta-analysis	Cu	In serum/plasma: AD (2929), HC (3547) In brain: AD (182), HC (166)		In brain: Cu↓ (SMD [95% CI]); −0.74 (−1.05, −0.43) In serum/plasma: Cu↑ 0.66 (0.34, 0.97)
[93]	CSF	GF-AAS	Fe	AD (16), MCI (17), FTD (22), HC (14)	69	Fe↑ (*p* < 0.001)
[94]	Plasma, erythrocytes	GF-AAS	Se	AD (34), HC (68)	102	Se↓ (in plasma and erythrocytes) (*p* < 0.001)
[95]	Plasma	Standard hospital assays	Mg	AD (1600), non-AD dementia (855), no dementia (100,193)	102,648	Both Mg↓ (multifactorial-adjusted HR [95% CI]; 1.5 [1.21–1.87]) and Mg↑ (1.34 [1.07–1.69]) associated with an increased risk of vascular-related non-AD dementia. There is no correlation observed for AD
[96]	CSF and plasma	Flame photometer measurement	Na	At risk for AD (43)	43	In CSF: Na↑ in high blood pressure patients at risk for AD (*p* < 0.01)
[97]	Serum	According to a photometric color	Mg	Dementia (2761), HC (42,698)	45,459	No difference
[98]	Serum	Routinely performed in hospital laboratories	K	AD (105), DLB (78)	183	K↑ predicts poorer cognitive prognosis for dementia patients (*p* = 0.003)
[99]	Plasma	ICP-MS	Cu, Zn	AD (95), HC (84)	179	No difference
[100]	Plasma	Total reflection X-ray fluorescence (TXRF) spectroscopy	Ca, Fe, Zn, Cu, Se, P	AD (44), HC (44)	88	Ca↑ (*p* = 0.025), P↑ (*p* = 1.33 × 10^−12^)
[101]	Blood (erythrocytes)	ICP-MS	Cu, Fe, Se	AD (32), HC (32)	64	Cu↑ (*p* < 0.001), Fe↑ (*p* < 0.001)
[102]	Serum	Ion-selective electrode method	Na, K	MCI (139), HC (371)	510	No difference
[62]	Urine and blood	ICP-MS	In blood: Cd, Pb, Hg, Se In urine: As	AD (170), HC (264)	434	No difference
[103]	Blood	AAS	Pb	AD (27), HC (54)	81	Pb↑ (*p* < 0.001)
[104]	CSF	ICP-MS	Ca	AD (45), HC (45)	90	No difference
[105]	Serum and urine	In serum: AAS In urine: GF-AAS	Cu	AD (385), HC (336), WD (9)	730	In serum: Cu↑ (*p* < 0.001) In urine: Cu↑ (*p* < 0.001)
[106]	Plasma	ICP-MS	Na, K, Ca, Mg, Fe, Zn, Cu, Se	AD (42), HC (43)	85	Zn↑ (in males) (*p* = 0.021)
[107]	Plasma	ICP-MS	Li, Mg, Al, Ca, Ti, V, Cr, Ca, Mn, Fe, Co, Ni, Cu, Zn, As, Se, Sr, Mo, Ba, Tl, Pb	AD (92), HC (161)	253	Al↑ (*p* < 0.001), Cu↑ (*p* < 0.001), Fe↑ (*p* < 0.001), Li↓ (*p* < 0.001), Mn↓ (*p* < 0.001), Zn↓ (*p* < 0.05)
[108]	Serum	Colorimetric endpoint method	Mg	Dementia (823, 662 of them had AD), no dementia (8746)	9569	Both Mg↓ (HR [95% CI]; 1.32 [1.02–1.69]) and Mg↑ (1.30 [1.02–1.67]) associated with an increased risk of dementia
[109]	Serum	AAS	Mg, Fe, Mn	AD (15), MCI (15), HC (15)	45	Mg↓ (*p* < 0.01), Mn↑ (*p* < 0.001)
[110]	Serum	Meta-analysis	Mn	AD (836), HC (1254)	2090	Mn↓ (SMD [95% CI]; −0.39 [−0.71, −0.08])
[111]	Circulatory (plasma/serum and blood), erythrocytes, CSF	Meta-analysis	Se	AD (594), HC (472)		Circulatory: Se↓ (SMD [95% CI]; −0.44 [−0.71, −0.17])
[112]	Serum	Photoelectric colorimetric assay	Cu, Fe, Zn	AD (125), HC (40)	165	Cu↑ (*p* = 0.014), Fe↑ (*p* = 0.027), Zn↓ (*p* = 0.020)
[113]	CSF, serum, erythrocytes	SEC-ICP-MS and tandem mass spectrometry	Se	CSF: AD (10), MCI (5), HC (31) Serum: AD (29), MCI (30), HC (30) Erythrocytes: AD (36), HC (39)	CSF: 46 Serum: 89 Erythrocytes: 75	In erythrocytes: Se↓ (*p* < 0.05)
[114]	Plasma	AAS	Se	AD (11), MCI (17), HC (12)	40	Se↓ (in AD, *p* = 0.049 and MCI, *p* = 0.003)
[115]	Brain and circulatory	Meta- analysis	Circulatory: Se Brain: Se, Zn	Circulatory: AD (660), HC (536) Brain: Se—AD (487), HC (353), Zn—AD (496), HC (306)		Circulatory: Se↓ (*p* < 0.05) Brain: no difference
[116]	Serum, CSF, and post-mortem brain tissue	ICP-MS	K and Rb	For serum: AD (171), MCI (128), HC (778) For CSF: AD (9), MCI (7), HC (36) For brain tissue: AD (30), HC (30)	For serum: 1077 For CSF: 52 For brain tissue: 60	In serum: K↑ (*p* < 0.05), Rb↓ (*p* < 0.001) In brain: K↓ (*p* < 0.01), Rb↓ (*p* < 0.001)
[117]	Serum	ICP-MS	Al, Sb, As, Be, Cd, Ca, Cr, Co, Cu, Fe, Pb, Hg, Mn, Mo, Ni, Se, Sr, Tl, Sn, U, V, and Zn	AD (34), MCI (20), SMC (24), HC (40)	118	Hg↓ (in AD, *p* < 0.001), Mn↓ (in AD, *p* < 0.001 and MCI, *p* = 0.024), Mo↑ (in AD, *p* = 0.001), Se↓ (in MCI, *p* = 0.015)
[118]	Serum, erythrocytes	ICP-MS	Pb, Mn	AD (206), MCI (129), HC (758)	1093	Mn↓ (in serum, *p* < 0.001)
[119]	Plasma	SEC-ICP-MS, solution nebulization (SN)-ICP-MS	Fe	AD (34), HC (36)	70	Fe↓ (*p* = 0.01)
[120]	Serum and hair	ICP-MS	Cu, Se, Zn, Mg, Mn, and Fe	AD (45), HC (33)	78	In serum: Mn↓ (*p* = 0.002) In hair: Se↓ (*p* = 0.005), Zn↓ (*p* = 0.02), Cu↑ (*p* = 0.013), Mn↑ (*p* = 0.009)
[121]	Serum	AAS (Cu, Mn) and Biorex diagnostics kit (Zn)	Cu, Mn, Zn	MCI (120), HC (120)	240	No difference
[122]	Serum	FAAS	Fe, Cu, Zn	AD (83), HC (83)	166	Cu↑ (*p* < 0.001), Fe↓ (*p* = 0.001)
		Meta- analysis		For Fe: AD (1084), HC (1319) For Zn: AD (862), HC (1705) For Cu: AD (1768), HC (2514)		Cu↑ (WMD = 10.474, *p* < 0.001), Zn↓ (WMD = −5.503; *p* < 0.001)
[123]	Serum, plasma, and CSF	Meta- analysis	Zn	For serum: AD (777), HC (1728) For plasma: AD (287), HC (166) For CSF: AD (292), HC (179)		In serum (plus in serum and plasma): Zn↓ (SMD [95% CI]; −0.46 [−0.76, −0.16])
[124]	Blood	ICP-MS	Cu, Se, Zn, Pb, and Hg	AD (15), MS (41), HC (23), healthy elderly controls (10)	89	Pb↓, Cu↓, Zn↓, Se↓ (for all comparisons *p* < 0.001)
[125]	Plasma	ICP-MS	Fe	AD (211), MCI (133), HC (768)	1112	Fe↓ (*p* = 0.049)
[126]	Plasma	GF-AAS	Se	AD (79), HC (93)	172	Se↓ (*p* < 0.001)
[127]	Serum	ICP-MS	Pb, Cd, Hg, As	AD (89), HC (118)	207	No difference
[128]	Serum	ICP-MS	Li, Al, V, Cr, Mn, Fe, Co, Cu, Zn, Se, Mo, Cd, and Pb	AD (30), MCI (16), HC (30)	76	Mn↓ (in AD and MCI), Al↑ (in AD and MCI), Se↓ (in AD and MCI), Fe↑ (in AD and MCI), Zn↓ (in AD)
[129]	Serum and CSF	Meta-analysis	Fe	AD (1813), HC (2401)	4214	In serum: Fe↓ (*p* < 0.001)
[130]	Erythrocytes and serum	ICP-MS	Zn	AD (205), MCI (126), HC (753)	1084	No difference
[131]	Blood and serum	ICP-MS (for Pb and Cd) and Gold amalgamation (for Hg)	Pb, Cd, and Hg	AD (80), HC (130)	210	No difference
[132]	CSF	ICP-MS	Cu, Fe, Mg, Mn, and Zn	AD (21), PD (20), ALS (52), HC (15)	108	Cu↑ (*p* < 0.01), Zn↑ (*p* < 0.01)
[133]	Serum, plasma, and CSF	Meta-analysis	Cu	Serum: AD (761), HC (664) Plasma: AD (205), HC (167) CSF: AD (116), HC (129)		In serum: Cu↑ (*p* = 0.001)
[134]	Plasma and CSF	ICP-MS	CSF/plasma quotients of Mg, Ca, Mn, Fe, Co, Ni, Cu, Zn, Se, Rb, Sr, Mo, Cd, Sn, Sb, Cs, Hg, and Pb	AD (264), HC (54)	318	CSF/plasma quotients of Mn↓ (*p* < 0.001), Rb↓ (*p* = 0.002), Sb↓ (*p* = 0.003), Pb↓ (*p* = 0.001), Hg↓ (*p* = 0.001), Co↑ (*p* < 0.001)
[135]	Serum	ICP-MS	Al, As, Cr, Co, Cu, I, Fe, Mn, Se, and Zn	AD (44), HC (41)	85	Zn↓ (*p* < 0.001)
[136]	CSF, plasma	ICP-MS	Mg, Ca, Mn, Fe, Cu, Zn, Rb, Sr, Cs	AD (174), AD with minor vascular components (90), DLB (29), HC (51)	344	In AD compared to LBD: CSF and plasma Mg↓ (*p* < 0.001), Ca↓ (*p* ≤ 0.001), Cu↓ (*p* ≤ 0.004), CSF Cs↓ (*p* < 0.001), plasma Zn↑ (*p* = 0.003) In AD compared to HC: No difference
[137]	CSF	AAS	Fe	AD (13), early stage of MCI (21), moderate MCI (10), HC (12)	56	No difference
[138]	Serum	HR-ICP-MS	Zn	AD (18), MCI (19), HC (16)	53	No difference
[139]	Plasma and CSF	ICP-MS	Mg, Ca, V, Mn, Fe, Co, Ni, Cu, Zn, Se, Rb, Sr, Mo, Cd, Sn, Sb, Cs, Hg, and Pb	AD (173), patients with a combination of AD and minor vascular components (AD + VaD; 87), HC (54)	314	In plasma: Mn↑ (*p* < 0.001), Hg↑ (*p* < 0.001), Co↓ (*p* < 0.01), Se↓ (*p* < 0.01), Cs↓ (*p* < 0.01) In CSF: V↓, Mn↓, Rb↓, Sb↓, Cs↓, Pb↓ (for all comparisons *p* < 0.001)
[140]	Serum	ICP-MS, ICP-AES	Ca, Cu, Fe, Mg, Si, Zn, Ba, Be, Bi, Cd, Hg, Li, Mo, Pb, Sb, Sn, Sr, Tl, W, Zr, Al, Co, Cr, Mn, Ni, and V	AD (53), PD (71), MS (60), HC (124)	308	Ca↑, Sn↑, Co↓, Fe↓, Zn↓ (for all comparisons *p* < 0.001)
[141]	Serum and whole blood	ICP-MS, ICP-AES	Al, Ba, Be, Bi, Ca, Cd, Co, Cr, Cu, Fe, Hg, Li, Mn, Mo, Ni, Pb, Sb, Si, Sn, Sr, Tl, V, W, Zn, and Zr	AD (60), HC (44)	104	In serum: Ca↑, Cd↑, Hg↑, Mg↑, Si↑, Sn↑, Al↓, Co↓, Fe↓, Zn↓ In blood: Cu↑, Li↑, Mn↑, Sn↑, Zr↑, Fe↓, Hg↓, Mo↓ (for all comparisons *p* ≤ 0.05)
[142]	Serum	Chromato-graphic or spectro- photometric methods	Fe, Zn, Mn, Se, Co, Cr, Cu, Mo, and AI	AD (8), VaD (8), cognitive impairment non-dementia (8), HC (11)	35	Se↓, Co↓, Cr↓, Cu↑, Al↑ (for all comparisons *p* < 0.001)
[143]	CSF, serum	AAS	Se	AD (27), HC (34)	61	No difference
[144]	Plasma	AAS	Cu	AD (44), HC (44)	88	No difference
[145]	Blood	AAS	Hg	AD (33), control group with major depression (45), control group with non-psychiatric disorders (65)	143	Hg↑ (*p* < 0.001)
[146]	Serum and CSF	AAS	Fe, Cu, Mn, and Zn	AD (26), HC (28)	54	CSF Zn↓ (*p* < 0.05)
[147]	Serum	AAS	Al	AD (17), HC (189), other dementias (15)	221	Al↑ (*p* = 0.001)
[148]	Whole blood	GF-AAS	Cd	AD (6), demented (10), HC (19)	35	No difference
[149]	CSF and serum	Ca was determined using the o-Cresol- phthalein method, whereas P was determined using the molybdate method	Ca and P	AD (40), multiple infarct dementia (25), aged controls (20), adult controls (20)	105	CSF Ca↓ (*p* < 0.01), P↓ (*p* < 0.01) (compared to adult controls)
[150]	CSF	AAS	Zn	AD (34), HC (34)	68	No difference
[151]	CSF	Inductively coupled argon plasma emission spectroscopy	Al, As, Ba, Be, Cd, Co, Cr, Cu, Fe, Mn, Mo, Ni, Pb, Se, Si, Sn, Ti, V, and Zn	AD (33), other dementia (16), no neurological disease (20)	69	Si↑ (*p* < 0.05), Zn↑ (*p* < 0.05)

Only studies conducted on humans were included in this table. (↓) decrease, (↑) increase. AAS, atomic absorption spectrophotometry; AD, Alzheimer’s disease; ALS, amyotrophic lateral sclerosis; AMD, age-related macular degeneration; CAA, cerebral amyloid angiopathy; CI, confidence interval; CSF, cerebrospinal fluid; DLB, dementia with Lewy bodies; FAAS, flame atomic absorption spectrometry; FTD, frontotemporal dementia; GF-AAS, graphite furnace atomic absorption spectrophotometry; HC, healthy controls; HR, hazard ratio; HR-ICP-MS, high resolution inductively coupled plasma mass spectrometry; ICP-AES, inductively coupled plasma atomic emission spectrometer; ICP-DRC-MS, inductively coupled plasma dynamic reaction cell mass spectrometry; ICP-MS, inductively coupled plasma mass spectrometry; ICP-OES, inductively coupled plasma optical emission spectroscopy; MCI, mild cognitive impairment; MS, multiple sclerosis; PD, Parkinson’s disease; SEC-ICP-MS, size exclusion chromatography inductively coupled plasma mass spectrometry; SMC, subjective memory complaint; SMD, standardized mean differences; VaD, vascular dementia; WD, Wilson disease; WMD, weighted mean difference.

**Table 2:** Correlation between metals and CSF protein AD biomarkers.

Reference	Analyzed Bodily Fluid	Measured Metals	Measured Biomarkers	Association of Metals with CSF Protein Biomarkers	Classification of Participants (Number of Patients)	Number of Participants
[74]	CSF and plasma	In CSF and plasma: As, B, Ca, Cd, Co, Cu, Fe, Hg, Li, Mg, Mn, Mo, Na, Ni, P, Pb, S, Se, Sr, Tl, Zn In CSF: Al, Ba, K	CSF Aβ_1–42_, t-tau, p-tau_181_, p-tau_231_, p-tau_199_, NFL, S100B, VILIP-1, YKL-40, PAPP-A, and albumin	Positive association of CSF heavy metals (As, Cd, Hg, Ni, Pb, and Tl), essential metals (Ca, Co, Cu, Fe, Mg, Mn, Mo, Na, K, and Zn), and essential nonmetals (P, S, and Se) and plasma Ni with CSF p-tau isoforms, VILIP-1, S100B, NFL, and YKL-40 (for all comparisons *p* ≤ 0.001)	CSF: AD (124), MCI (50), HC (19) Plasma: AD (93), MCI (35), HC (15)	CSF: 193 Plasma: 143
[185]	Serum and blood	Blood Se	Serum Aβ_1–40_ and Aβ_1–42_	Negative association of Se with Aβ_1–40_ and Aβ_1–42_ and positive association with Aβ_1–42_/Aβ_1–40_ ratio (*p* < 0.05)	Elderly individuals	469
[90]	CSF	Ni, Cr, Zn, Mn, Co, and Cu	Aβ_1–42_, Aβ_1–40_, t-tau, p-tau_181_, NFL	The negative correlation of Fe and ferritin with Aβ_1–42_ (r = −0.506, *p* < 0.001)	AD (20), cerebral amyloid angiopathy (10), controls (10)	40
[186]	Serum, CSF	Serum Ca	CSF Aβ_1–42_, t-tau, p-tau_181_	Serum Ca negatively correlated with CSF Aβ_1–42_ (β = −0.558, *p* = 0.008)	MCI (811), cognitively normal (413)	1224
[187]	Serum	Al, Pb, Mn, and Zn	T-tau	The negative correlation between Mn and t-tau (r = −0.341, *p* = 0.003)	Aluminum foundry workers (75), non-occupationally exposed subjects as controls (75)	150
[188]	CSF	Fe, Cr, Mn, Ni, Cu, Zn	Aβ_1–42_, t-tau, p-tau, and CSF/serum albumin ratio	Positive correlation of Fe and Cu with Aβ_1–42_ (β_Fe_ = 0.21, *p* = 0.004, β_Cu_ = 0.23, *p* = 0.001), t-tau (β_Fe_ = 0.27, *p* < 0.001, β_Cu_ = 0.23, *p* = 0.001), p-tau (β_Fe_ = 0.30, *p* < 0.001, β_Cu_ = 0.26, *p* < 0.001) and CSF/serum albumin ratio (β_Fe_ = 0.52, *p* < 0.001, β_Cu_ = 0.65, *p* < 0.001). Positive correlation of Zn with CSF/serum albumin ratio (β = 0.17, *p* = 0.02).	AD (85), MCI (72), subjective cognitive impairment (32), VaD (7)	196
[104]	CSF	Ca	Aβ_1–42_, t-tau, p-tau_181_	No association of CSF Ca with CSF AD biomarkers	AD (45), HC (45)	90
[189]	CSF	Se	CSF Aβ_1–42_, t-tau, and p-tau	The negative correlation between Se and Aβ_1–42_ (β = −0.27, 95% CI; −0.66–0.11)	MCI (56), during the 42 months, 21 developed AD, 4 FTD, and 2 DLB	56
[190]	Blood, plasma	Blood Se	Plasma Aβ_1–42_, t-tau	No association of Se with plasma AD biomarkers	AD (30), VaD (35), HC (40)	105
[191]	Blood and plasma	Blood Mn	Plasma Aβ_1–40_ and Aβ_1–42_	Positive correlation of Mn with Aβ_1–40_ (R^2^ = 0.127, *p* = 0.024) and Aβ_1–42_ (R^2^ = 0.163, *p* = 0.010)	AD (20), MCI (10), HC (10)	40
[192]	CSF	Mg, Ca, V, Cd, Sn, Sb, Mn, Ni, Cu, Zn, Se, Rb, Fe, Co, Sr, Mo, Cs, Hg, and Pb	Aβ_1–42_, t-tau, p-tau_181_	Positive correlation between Mn and t-tau (r_s_ = 0.22, *p* = 0.004) and p-tau_181_ (r_s_ = 0.18, *p* = 0.021). The negative correlation of Cs with t-tau (r_s_ = −0.49, *p* = 0.026), and a positive correlation of Cs with Aβ_1–42_ (r_s_ = 0.49, *p* = 0.026).	AD (173), AD + minor vascular components (87), HC (54)	314
[193]	CSF (taken from brain ventricles)	Cu, Zn, Fe, Mn, and Cr	Aβ_1–42_	The negative correlation of Cu (β coefficient = −1.3, *p* < 0.001), Zn (β coefficient = −1.26, *p* < 0.001), Fe (*p* = 0.001), Mn (*p* = 0.003), and Cr (*p* = 0.01) with Aβ_1–42_	AD (25), VaD (18), other dementias (6), clinically non-demented individuals (82)	131
[194]	CSF	Cu intake	Aβ_1–42_, t-tau, p-tau_181_	Cu intake did not affect the t-tau and p-tau_181_ levels, but the Aβ_1–42_ levels decreased by 30% in the placebo group and only by 10% in the verum group	AD (68)	68
[195]	CSF, serum	Serum K	CSF Aβ_1–42_	Low serum K in mid-life, but not late life, is associated with low CSF Aβ_1–42_ in late life (β = 153.9, *p* = 0.041)	Women from Goteborg	1080
[196]	Serum, plasma and CSF	Cu (in serum)	Aβ_1–42_, t-tau (in CSF)	The negative correlation of Cu with Aβ_1–42_ (r = −0.46, *p* = 0.002), and the positive correlation of Cu with t-tau (r = 0.4, *p* = 0.03)	AD (28), HC (25)	53
[145]	Blood and CSF	Hg (in the blood)	Aβ_1–42_ (in CSF)	Positive correlation between Hg and Aβ_1–42_ (r = 0.744, *p* < 0.001)	AD (33), age-matched control patients with major depression (45), and a control group of patients with a variety of non-psychiatric disorders (65) served as comparison groups	143

This table only included research conducted on human subjects. Aβ_1–42_, amyloid β_1–42_; AD, Alzheimer’s disease; CSF, cerebrospinal fluid; DLB, dementia with Lewy bodies; FTD, frontotemporal dementia; HC, healthy control; MCI, mild cognitive impairment; NFL, neurofilament light chain; PAPP-A, pregnancy-associated plasma protein A; p-tau_181_, tau protein phosphorylated at Thr 181; p-tau_231_, tau protein phosphorylated at threonine 231; p-tau_199_, tau protein phosphorylated at serine 199; S100B, S100 calcium-binding protein B; t-tau, total tau; VaD, vascular dementia; VILIP-1, visinin-like protein 1; YKL-40, chitinase-3-like protein 1.

**Table 3:** Mendelian randomization studies that investigated the role of metals in AD.

Reference	MR Analysis	Measured Metals	Observed Association
[218]	Two-sample MR	Mg, Ca, Fe, Cu, Zn, Se, P	Higher Cu levels as a protective factor for AD risk
[170]	Two-sample MR	Pb (in the blood)	Higher Pb levels as a risk factor for AD
[241]	Two-sample MR	Ca (in serum)	Higher Ca levels as a protective factor for AD risk
[243]	MR	Ca (in serum)	Higher Ca levels as a protective factor for AD risk
[219]	Two-sample MR	Cu, Zn, Fe	No association
[220]	Two-sample MR	Ca, Mg, Fe, Cu, Zn (in the blood)	Higher Cu levels as a protective factor for AD risk

AD, Alzheimer’s disease; MR, Mendelian randomization.

## Data Availability

Not applicable.

## Abbreviations

AAS, atomic absorption spectrophotometry; Aβ, amyloid β; Aβ_1–40_, amyloid β_1–40_; Aβ_1–42_, amyloid β_1–42_; AD, Alzheimer’s disease; Al, aluminum; ALS, amyotrophic lateral sclerosis; AMD, age-related macular degeneration; ApoE, apolipoprotein E; APP, amyloid precursor protein; As, arsenic; BBB, blood-brain barrier; Ca, calcium; CAA, cerebral amyloid angiopathy; Cd, cadmium; CI, confidence interval; Co, cobalt; CQ, clioquinol; Cr, chromium; Cs, cesium; CSF, cerebrospinal fluid; Cu, copper; DLB, dementia with Lewy bodies; fAD, familial AD; FAAS, flame atomic absorption spectrometry; Fe, iron; FTD, frontotemporal dementia; GF-AAS, graphite furnace atomic absorption spectrophotometry; HC, healthy controls; Hg, mercury; HR, hazard ratio; HR-ICP-MS, high resolution inductively coupled plasma mass spectrometry; ICP-AES, inductively coupled plasma atomic emission spectrometer; ICP-DRC-MS, inductively coupled plasma dynamic reaction cell mass spectrometry; ICP-MS, inductively coupled plasma mass spectroscopy; K, potassium; Li, lithium; MCI, mild cognitive impairment; Mg, magnesium; Mn, manganese; Mo, molybdenum; MR, Mendelian randomization; MS, multiple sclerosis; Na, sodium; NFL, neurofilament light chain; NFT, neurofibrillary tangles; Ni, nickel; NMDA, *N*-methyl-D-aspartate; P, phosphorus; PAPP-A, pregnancy-associated plasma protein A, pappalysin-1; Pb, lead; PD, Parkinson’s disease; p-tau_181_, tau phosphorylated at Thr 181; p-tau_231_, tau phosphorylated at Thr 231; p-tau_199_, tau phosphorylated at Ser 199; S, sulfur; Se, selenium; SEC-ICP-MS, size exclusion chromatography inductively coupled plasma mass spectrometry; SMC, subjective memory complaint; SMD, standardized mean differences; SP, senile plaques; S100B, S100 calcium-binding protein B; t-tau, total tau; VaD, vascular dementia; VILIP-1, visinin-like protein 1; WD, Wilson disease; WMD, weighted mean difference; YKL-40, chitinase-3-like protein 1; Zn, zinc.
